# Supplementary material for: Sex differences in the prevalence of metabolic syndrome and associated factors in the general population of Mongolia: A nationwide study
Source: PLoS One. 2024 Oct 23;19(10):e0311320. doi: 10.1371/journal.pone.0311320 (PMC11498733; doi:10.1371/journal.pone.0311320)
Supplement: S1 Table — (DOCX) [file pone.0311320.s001.docx]

**S1 Table. Characteristics of participants according to residence (N = 5,695).**

| **Variables** | **Total (N = 5695)** | **Rural (N = 2039)** | **Urban (N = 3656)** | **P-value^c^** |
| --- | --- | --- | --- | --- |
|  | N (%) | n (%) | n (%) |  |
| **Sex** | | | | <0.001 |
| Male | 2577 (45.3) | 1015 (49.8) | 1562 (42.7) |  |
| Female | 3118 (54.7) | 1024 (50.2) | 2094 (57.3) |  |
| **Age group (years)** | | | | 0.104 |
| 18-29 | 1085 (19.1) | 417 (20.5) | 668 (18.3) |  |
| 30-44 | 2167 (38.1) | 751 (36.8) | 1416 (38.7) |  |
| 45-69 | 2443 (42.9) | 871 (42.7) | 1572 (43.0) |  |
| **Ethnicity (N = 5669)** | | | | <0.001 |
| Khalkh | 4807 (84.8) | 1583 (77.9) | 3224 (88.7) |  |
| Kazakh | 174 (3.1) | 96 (4.7) | 78 (2.1) |  |
| Durvud | 240 (4.2) | 103 (5.1) | 137 (3.8) |  |
| Buryat | 157 (2.8) | 99 (4.9) | 58 (1.6) |  |
| Other | 291 (5.1) | 152 (7.5) | 139 (3.8) |  |
| **Region** | | | | <0.001 |
| Western region | 717 (12.6) | 447 (21.9) | 270 (7.4) |  |
| Eastern region | 592 (10.4) | 379 (18.6) | 213 (5.8) |  |
| Khangai region | 1094 (19.2) | 667 (32.7) | 427 (11.7) |  |
| Central region | 921 (16.2) | 546 (26.8) | 375 (10.3) |  |
| Ulaanbaatar | 2371 (41.6) | 0 (0.0) | 2371 (64.9) |  |
| **Education (N = 5694)** | | | | <0.001 |
| None | 218 (3.8) | 138 (6.8) | 80 (2.2) |  |
| Primary | 344 (6.0) | 233 (11.4) | 111 (3.0) |  |
| Secondary | 2567 (45.1) | 1079 (52.9) | 1488 (40.7) |  |
| College ≤ | 2565 (45.0) | 589 (28.9) | 1976 (54.1) |  |
| **Marital status (N = 5688)** | | | | 0.048 |
| Never married | 941 (16.5) | 333 (16.4) | 608 (16.6) |  |
| Married | 4160 (73.1) | 1519 (74.6) | 2641 (72.3) |  |
| Other^a^ | 587 (10.3) | 184 (9.0) | 403 (11.0) |  |
| **Employment (N = 5575)** | | | | <0.001 |
| Full-time | 2134 (38.3) | 545 (27.3) | 1589 (44.4) |  |
| Part-time | 1743 (31.3) | 892 (44.6) | 851 (23.8) |  |
| Unemployed^b^ | 1698 (30.5) | 562 (28.1) | 1136 (31.8) |  |
| **Monthly income (×1000 MNT)** | | | | <0.001 |
| <100 | 872 (15.3) | 518 (25.4) | 354 (9.7) |  |
| 100-<300 | 641 (11.3) | 349 (17.1) | 292 (8.0) |  |
| 300-<500 | 527 (9.3) | 226 (11.1) | 301 (8.2) |  |
| 500-<1000 | 2235 (39.2) | 676 (33.2) | 1559 (42.6) |  |
| 1000≤ | 1420 (24.9) | 270 (13.2) | 1150 (31.5) |  |
| **Currently smoking** | | | | 0.892 |
| No | 4243 (74.5) | 1517 (74.4) | 2726 (74.6) |  |
| Yes | 1452 (25.5) | 522 (25.6) | 930 (25.4) |  |
| **Currently drinking** | | | | 0.002 |
| No | 3551 (62.4) | 1216 (59.6) | 2335 (63.9) |  |
| Yes | 2144 (37.6) | 823 (40.4) | 1321 (36.1) |  |
| **Insufficient fruit and vegetable intake (N = 5455)** | | | | <0.001 |
| No | 1614 (29.6) | 407 (20.9) | 1207 (34.4) |  |
| Yes | 3841 (70.4) | 1538 (79.1) | 2303 (65.6) |  |
| **Level of physical activity (N = 5607)** | | | | <0.001 |
| High | 1450 (25.9) | 728 (36.5) | 722 (20.0) |  |
| Moderate | 2464 (43.9) | 782 (39.2) | 1682 (46.6) |  |
| Low | 1693 (30.2) | 486 (24.3) | 1207 (33.4) |  |
| **Sedentary behavior** | | | | <0.001 |
| No | 5270 (92.5) | 1944 (95.3) | 3326 (91.0) |  |
| Yes | 425 (7.5) | 95 (4.7) | 330 (9.0) |  |
| **History of HT** | | | | 0.043 |
| No | 3865 (67.9) | 1418 (69.5) | 2447 (66.9) |  |
| Yes | 1830 (32.1) | 621 (30.5) | 1209 (33.1) |  |
| **History of DM** | | | | <0.001 |
| No | 5397 (94.8) | 1959 (96.1) | 3438 (94.0) |  |
| Yes | 298 (5.2) | 80 (3.9) | 218 (6.0) |  |
| **History of HCE** | | | | <0.001 |
| No | 5333 (93.6) | 1968 (96.5) | 3365 (92.0) |  |
| Yes | 362 (6.4) | 71 (3.5) | 291 (8.0) |  |
| **History of CVD** | | | | 0.006 |
| No | 4757 (83.5) | 1740 (85.3) | 3017 (82.5) |  |
| Yes | 938 (16.5) | 299 (14.7) | 639 (17.5) |  |
| **Body mass index (N = 5694)** | | | | 0.295 |
| Normal | 2190 (38.5) | 816 (40.0) | 1374 (37.6) |  |
| Underweight | 137 (2.4) | 49 (2.4) | 88 (2.4) |  |
| Overweight | 2041 (35.8) | 703 (34.5) | 1338 (36.6) |  |
| Obesity | 1326 (23.3) | 471 (23.1) | 855 (23.4) |  |
| **Abdominal obesity** |  |  |  | <0.001 |
| No | 2110 (37.1) | 831 (40.8) | 1279 (35.0) |  |
| Yes | 3585 (62.9) | 1208 (59.2) | 2377 (65.0) |  |

MNT, Mongolian tugrik; HT, hypertension; DM, diabetes mellitus; HCE, hypercholesterolemia; CVD, cardiovascular disease.

^a^Other includes divorced, widowed, and separated.

^b^Unemployed includes a student, a retired person, and an unemployed person.

^c^A chi-square test was performed.

1 USD = 3,481.66 MNT on April 30, 2023.
