# Supplementary material for: Sex differences in the prevalence of metabolic syndrome and associated factors in the general population of Mongolia: A nationwide study
Source: PLoS One. 2024 Oct 23;19(10):e0311320. doi: 10.1371/journal.pone.0311320 (PMC11498733; doi:10.1371/journal.pone.0311320)
Supplement: S2 Table — (DOCX) [file pone.0311320.s002.docx]

**S2 Table. Characteristics of participants according to body mass index (N = 5,694).**

| **Variables** | **Total**  **(N = 5694)** | **Body mass index** | | | | **P-value^c^** |
| --- | --- | --- | --- | --- | --- | --- |
|  |  | **Normal**  **(N = 2190)** | **Underweight**  **(N = 137)** | **Overweight**  **(N = 2041)** | **Obesity**  **(N = 1326)** |  |
|  | N (%) | n (%) | n (%) | n (%) | n (%) |  |
| **Sex** | | | | | | <0.001 |
| Male | 2577 (45.3) | 1070 (48.9) | 71 (51.8) | 927 (45.5) | 509 (38.4) |  |
| Female | 3117 (54.7) | 1120 (51.1) | 66 (48.2) | 1114 (54.6) | 817 (61.6) |  |
| **Age group (years)** | | | | | | <0.001 |
| 18-29 | 1085 (19.1) | 649 (29.6) | 73 (53.3) | 254 (12.4) | 109 (8.2) |  |
| 30-44 | 2167 (38.1) | 830 (37.9) | 27 (19.7) | 818 (40.1) | 492 (37.1) |  |
| 45-69 | 2442 (42.8) | 711 (32.5) | 37 (27.0) | 969 (47.5) | 725 (54.7) |  |
| **Ethnicity (N=5668)** | | | | | | <0.001 |
| Khalkh | 4806 (84.8) | 1803 (82.9) | 110 (80.3) | 1748 (86.1) | 1145 (86.4) |  |
| Kazakh | 174 (3.1) | 90 (4.1) | 12 (8.8) | 54 (2.7) | 18 (1.4) |  |
| Durvud | 240 (4.2) | 106 (4.9) | 2 (1.5) | 79 (3.9) | 53 (4.0) |  |
| Buryat | 157 (2.8) | 53 (2.4) | 6 (4.4) | 52 (2.6) | 46 (3.5) |  |
| Other | 291 (5.1) | 123 (5.7) | 7 (5.1) | 98 (4.8) | 63 (4.8) |  |
| **Residence** | | | | | | 0.295 |
| Rural | 2039 (35.8) | 816 (37.3) | 49 (35.8) | 703 (34.4) | 471 (35.5) |  |
| Urban | 3655 (64.2) | 1374 (62.7) | 88 (64.2) | 1338 (65.6) | 855 (64.5) |  |
| **Region** | | | | | | <0.001 |
| Western region | 717 (12.6) | 327 (14.9) | 18 (13.1) | 235 (11.5) | 137 (10.3) |  |
| Eastern region | 592 (10.4) | 241 (11.0) | 18 (13.1) | 210 (10.3) | 123 (9.3) |  |
| Khangai region | 1093 (19.2) | 445 (20.3) | 16 (11.7) | 392 (19.2) | 240 (18.1) |  |
| Central region | 921 (16.2) | 311 (14.2) | 23 (16.8) | 330 (16.2) | 257 (19.4) |  |
| Ulaanbaatar | 2371 (41.6) | 866 (39.5) | 62 (45.3) | 874 (42.8) | 569 (42.9) |  |
| **Education (N = 5693)** | | | | | | <0.001 |
| None | 218 (3.8) | 122 (5.6) | 5 (3.6) | 50 (2.4) | 41 (3.1) |  |
| Primary | 344 (6.0) | 137 (6.3) | 5 (3.6) | 132 (6.5) | 70 (5.3) |  |
| Secondary | 2567 (45.1) | 1001 (45.7) | 70 (51.1) | 902 (44.2) | 594 (44.8) |  |
| College ≤ | 2564 (45.1) | 930 (42.5) | 57 (41.6) | 957 (46.9) | 620 (46.8) |  |
| **Marital status (N = 5687)** | | | | | | <0.001 |
| Never married | 941 (16.5) | 521 (23.8) | 55 (40.4) | 230 (11.3) | 135 (10.2) |  |
| Married | 4159 (73.1) | 1455 (66.5) | 70 (51.5) | 1589 (77.9) | 1045 (78.9) |  |
| Other^a^ | 587 (10.4) | 212 (9.7) | 11 (8.1) | 220 (10.8) | 144 (10.9) |  |
| **Employment (N = 5574)** | | | | | | <0.001 |
| Full-time | 2134 (38.3) | 764 (35.7) | 42 (31.8) | 810 (40.4) | 518 (39.8) |  |
| Part-time | 1743 (31.3) | 711 (33.3) | 36 (27.3) | 628 (31.3) | 368 (28.3) |  |
| Unemployed^b^ | 1697 (30.4) | 663 (31.0) | 54 (40.9) | 566 (28.2) | 414 (31.8) |  |
| **Monthly income (×1000 MNT)** | | | | | | <0.001 |
| <100 | 872 (15.3) | 423 (19.3) | 23 (16.8) | 239 (11.7) | 187 (14.1) |  |
| 100-<300 | 641 (11.3) | 263 (12.0) | 22 (16.1) | 230 (11.3) | 126 (9.5) |  |
| 300-<500 | 527 (9.3) | 180 (8.2) | 7 (5.1) | 205 (10.0) | 135 (10.2) |  |
| 500-<1000 | 2234 (39.2) | 820 (37.4) | 46 (33.6) | 821 (40.2) | 547 (41.3) |  |
| 1000≤ | 1420 (24.9) | 504 (23.0) | 39 (28.5) | 546 (26.8) | 331 (25.0) |  |
| **Currently smoking** | | | | | | <0.001 |
| No | 4242 (74.5) | 1553 (70.9) | 101 (73.7) | 1544 (75.6) | 1044 (78.7) |  |
| Yes | 1452 (25.5) | 637 (29.1) | 36 (26.3) | 497 (24.4) | 282 (21.3) |  |
| **Currently drinking** | | | | | | 0.023 |
| No | 3551 (62.4) | 1378 (62.9) | 101 (73.7) | 1244 (61.0) | 828 (62.4) |  |
| Yes | 2143 (37.6) | 812 (37.1) | 36 (26.3) | 797 (39.0) | 498 (37.6) |  |
| **Insufficient fruit and vegetable intake (N = 5454)** | | | | | | 0.597 |
| No | 1613 (29.6) | 607 (29.0) | 40 (30.3) | 596 (30.7) | 370 (28.8) |  |
| Yes | 3841 (70.4) | 1487 (71.0) | 92 (69.7) | 1347 (69.3) | 915 (71.2) |  |
| **Level of physical activity (N = 5606)** | | | | | | <0.001 |
| High | 1450 (25.9) | 669 (31.0) | 27 (19.9) | 488 (24.3) | 266 (20.4) |  |
| Moderate | 2463 (43.9) | 947 (43.9) | 68 (50.0) | 902 (44.9) | 546 (41.9) |  |
| Low | 1693 (30.2) | 541 (25.1) | 41 (30.1) | 620 (30.8) | 491 (37.7) |  |
| **Sedentary behavior** | | | | | | 0.145 |
| No | 5269 (92.5) | 2035 (92.9) | 126 (92.0) | 1900 (93.1) | 1208 (91.1) |  |
| Yes | 425 (7.5) | 155 (7.1) | 11 (8.0) | 141 (6.9) | 118 (8.9) |  |
| **History of HT** | | | | | | <0.001 |
| No | 3865 (67.9) | 1742 (79.5) | 112 (81.8) | 1343 (65.8) | 668 (50.4) |  |
| Yes | 1829 (32.1) | 448 (20.5) | 25 (18.2) | 698 (34.2) | 658 (49.6) |  |
| **History of DM** | | | | | | <0.001 |
| No | 5396 (94.8) | 2130 (97.3) | 136 (99.3) | 1933 (94.7) | 1197 (90.3) |  |
| Yes | 298 (5.2) | 60 (2.7) | 1 (0.7) | 108 (5.3) | 129 (9.7) |  |
| **History of HCE** | | | | | | <0.001 |
| No | 5333 (93.7) | 2122 (96.9) | 136 (99.3) | 1907 (93.4) | 1168 (88.1) |  |
| Yes | 361 (6.3) | 68 (3.1) | 1 (0.7) | 134 (6.6) | 158 (11.9) |  |
| **History of CVD** | | | | | | 0.095 |
| No | 4756 (83.5) | 1850 (84.5) | 110 (80.3) | 1714 (84.0) | 1082 (81.6) |  |
| Yes | 938 (16.5) | 340 (15.5) | 27 (19.7) | 327 (16.0) | 244 (18.4) |  |
| **Abdominal obesity** | | | | | | <0.001 |
| No | 2110 (37.1) | 1602 (73.2) | 124 (90.5) | 355 (17.4) | 29 (2.2) |  |
| Yes | 3584 (62.9) | 588 (26.8) | 13 (9.5) | 1686 (82.6) | 1297 (97.8) |  |

MNT, Mongolian tugrik; HT, hypertension; DM, diabetes mellitus; HCE, hypercholesterolemia; CVD, cardiovascular disease.

^a^Other includes divorced, widowed, and separated.

^b^Unemployed includes a student, a retired person, and an unemployed person.

^c^A chi-square test was performed.

1 USD = 3,481.66 MNT on April 30, 2023.
