# Supplementary material for: Sex differences in the prevalence of metabolic syndrome and associated factors in the general population of Mongolia: A nationwide study
Source: PLoS One. 2024 Oct 23;19(10):e0311320. doi: 10.1371/journal.pone.0311320 (PMC11498733; doi:10.1371/journal.pone.0311320)
Supplement: S3 Table — (DOCX) [file pone.0311320.s003.docx]

**S3 Table. Characteristics of participants according to abdominal obesity (N = 5,695).**

| **Variables** | **Total (N = 5695)** | **Abdominal obesity** | | **P-value^c^** |
| --- | --- | --- | --- | --- |
|  |  | **Yes (N = 3585)** | **No (N = 2110)** |  |
|  | N (%) | n (%) | n (%) |  |
| **Sex** | | | | <0.001 |
| Male | 2577 (45.3) | 1272 (35.5) | 1305 (61.8) |  |
| Female | 3118 (54.7) | 2313 (64.5) | 805 (38.2) |  |
| **Age group (years)** | | | | <0.001 |
| 18-29 | 1085 (19.1) | 405 (11.3) | 680 (32.2) |  |
| 30-44 | 2167 (38.1) | 1384 (38.6) | 783 (37.1) |  |
| 45-69 | 2443 (42.9) | 1796 (50.1) | 647 (30.7) |  |
| **Ethnicity (N=5669)** | | | | 0.005 |
| Khalkh | 4807 (84.8) | 3064 (85.8) | 1743 (83.2) |  |
| Kazakh | 174 (3.1) | 87 (2.4) | 87 (4.2) |  |
| Durvud | 240 (4.2) | 148 (4.1) | 92 (4.4) |  |
| Buryat | 157 (2.8) | 98 (2.7) | 59 (2.8) |  |
| Other | 291 (5.1) | 176 (4.9) | 115 (5.5) |  |
| **Residence** | | | | <0.001 |
| Rural | 2039 (35.8) | 1208 (33.7) | 831 (39.4) |  |
| Urban | 3656 (64.2) | 2377 (66.3) | 1279 (60.6) |  |
| **Region** | | | | <0.001 |
| Western region | 717 (12.6) | 447 (12.5) | 270 (12.8) |  |
| Eastern region | 592 (10.4) | 332 (9.3) | 260 (12.3) |  |
| Khangai region | 1094 (19.2) | 620 (17.3) | 474 (22.5) |  |
| Central region | 921 (16.2) | 601 (16.8) | 320 (15.2) |  |
| Ulaanbaatar | 2371 (41.6) | 1585 (44.2) | 786 (37.3) |  |
| **Education (N = 5694)** | | | | <0.001 |
| None | 218 (3.8) | 115 (3.2) | 103 (4.9) |  |
| Primary | 344 (6.0) | 192 (5.4) | 152 (7.2) |  |
| Secondary | 2567 (45.1) | 1580 (44.1) | 987 (46.8) |  |
| College ≤ | 2565 (45.0) | 1697 (47.3) | 868 (41.1) |  |
| **Marital status (N = 5688)** | | | | <0.001 |
| Never married | 941 (16.5) | 389 (10.9) | 552 (26.2) |  |
| Married | 4160 (73.1) | 2806 (78.4) | 1354 (64.3) |  |
| Other^a^ | 587 (10.3) | 386 (10.8) | 201 (9.5) |  |
| **Employment (N = 5575)** | | | | <0.001 |
| Full-time | 2134 (38.3) | 1391 (39.6) | 743 (36.1) |  |
| Part-time | 1743 (31.3) | 1007 (28.7) | 736 (35.7) |  |
| Unemployed^b^ | 1698 (30.5) | 1116 (31.8) | 582 (28.2) |  |
| **Monthly income (×1000 MNT)** | | | | <0.001 |
| <100 | 872 (15.3) | 454 (12.7) | 418 (19.8) |  |
| 100-<300 | 641 (11.3) | 389 (10.9) | 252 (11.9) |  |
| 300-<500 | 527 (9.3) | 364 (10.2) | 163 (7.7) |  |
| 500-<1000 | 2235 (39.2) | 1452 (40.5) | 783 (37.1) |  |
| 1000≤ | 1420 (24.9) | 926 (25.8) | 494 (23.4) |  |
| **Currently smoking** | | | | <0.001 |
| No | 4243 (74.5) | 2843 (79.3) | 1400 (66.4) |  |
| Yes | 1452 (25.5) | 742 (20.7) | 710 (33.6) |  |
| **Currently drinking** | | | | 0.021 |
| No | 3551 (62.4) | 2276 (63.5) | 1275 (60.4) |  |
| Yes | 2144 (37.6) | 1309 (36.5) | 835 (39.6) |  |
| **Insufficient fruit and vegetable intake (N = 5455)** | | | | 0.093 |
| No | 1614 (29.6) | 1044 (30.4) | 570 (28.2) |  |
| Yes | 3841 (70.4) | 2392 (69.6) | 1449 (71.8) |  |
| **Level of physical activity (N = 5607)** | | | | <0.001 |
| High | 1450 (25.9) | 755 (21.4) | 695 (33.4) |  |
| Moderate | 2464 (43.9) | 1581 (44.8) | 883 (42.5) |  |
| Low | 1693 (30.2) | 1191 (33.8) | 502 (24.1) |  |
| **Sedentary behavior** | | | | 0.193 |
| No | 5270 (92.5) | 3305 (92.2) | 1965 (93.1) |  |
| Yes | 425 (7.5) | 280 (7.8) | 145 (6.9) |  |
| **History of HT** | | | | <0.001 |
| No | 3865 (67.9) | 2147 (59.9) | 1718 (81.4) |  |
| Yes | 1830 (32.1) | 1438 (40.1) | 392 (18.6) |  |
| **History of DM** | | | | <0.001 |
| No | 5397 (94.8) | 3333 (93.0) | 2064 (97.8) |  |
| Yes | 298 (5.2) | 252 (7.0) | 46 (2.2) |  |
| **History of HCE** | | | | <0.001 |
| No | 5333 (93.6) | 3276 (91.4) | 2057 (97.5) |  |
| Yes | 362 (6.4) | 309 (8.6) | 53 (2.5) |  |
| **History of CVD** | | | | 0.006 |
| No | 4757 (83.5) | 2954 (82.4) | 1803 (85.5) |  |
| Yes | 938 (16.5) | 631 (17.6) | 307 (14.5) |  |
| **Body mass index (N = 5694)** | | | | <0.001 |
| Normal | 2190 (38.5) | 588 (16.4) | 1602 (75.9) |  |
| Underweight | 137 (2.4) | 13 (0.4) | 124 (5.9) |  |
| Overweight | 2041 (35.8) | 1686 (47.0) | 355 (16.8) |  |
| Obesity | 1326 (23.3) | 1297 (36.2) | 29 (1.4) |  |

MNT, Mongolian tugrik; HT, hypertension; DM, diabetes mellitus; HCE, hypercholesterolemia; CVD, cardiovascular disease.

^a^Other includes divorced, widowed, and separated.

^b^Unemployed includes a student, a retired person, and an unemployed person.

^c^A chi-square test was performed.

1 USD = 3,481.66 MNT on April 30, 2023.
