# Supplementary material for: Sex differences in the prevalence of metabolic syndrome and associated factors in the general population of Mongolia: A nationwide study
Source: PLoS One. 2024 Oct 23;19(10):e0311320. doi: 10.1371/journal.pone.0311320 (PMC11498733; doi:10.1371/journal.pone.0311320)
Supplement: S4 Table — (DOCX) [file pone.0311320.s004.docx]

**S4 Table. Factors associated with metabolic syndrome among all participants (N = 5,695).**

| **Variables** | **Model 1** | | **Model 2** | | **Model 3** | |
| --- | --- | --- | --- | --- | --- | --- |
|  | **AOR**  **(95% CI)** | **P-value** | **AOR**  **(95% CI)** | **P-value** | **AOR**  **(95% CI)** | **P-value** |
| **Age group (years)** | | | | | | |
| 18-29 | 1  (Reference) |  | 1  (Reference) |  | 1  (Reference) |  |
| 30-45 | 1.47  (1.16-1.87) | 0.002 | 1.58  (1.27-1.97) | <0.001 | 1.54  (1.24-1.92) | <0.001 |
| 45-69 | 2.24  (1.76-2.86) | <0.001 | 2.42  (1.94-3.02) | <0.001 | 2.37  (1.90-2.95) | <0.001 |
| **Ethnicity** | | | | | | |
| Khalkh | 1  (Reference) |  | 1  (Reference) |  | 1  (Reference) |  |
| Kazak | 1.47  (0.92-2.33) | 0.107 | 1.49  (0.94-2.35) | 0.087 | 1.56  (0.98-2.47) | 0.061 |
| Durvud | 0.75  (0.52-1.09) | 0.133 | 0.73  (0.51-1.06) | 0.101 | 0.75  (0.52-1.08) | 0.126 |
| Buriad | 1.88  (1.20-2.95) | 0.006 | 1.99  (1.28-3.10) | 0.002 | 1.99  (1.28-3.10) | 0.002 |
| Other | 1.01  (0.72-1.42) | 0.951 | 1.01  (0.72-1.42) | 0.949 | 1.01  (0.72-1.42) | 0.967 |
| **Region** | | | | | | |
| Western | 1  (Reference) |  | 1  (Reference) |  | 1  (Reference) |  |
| Eastern | 0.86  (0.61-1.21) | 0.392 | 0.88  (0.63-1.23) | 0.468 | 0.89  (0.64-1.24) | 0.495 |
| Khangai | 1.05  (0.78-1.40) | 0.768 | 1.04  (0.78-1.40) | 0.777 | 1.06  (0.79-1.42) | 0.703 |
| Central | 1.37  (1.01-1.86) | 0.042 | 1.36  (1.01-1.83) | 0.045 | 1.39  (1.03-1.88) | 0.031 |
| Ulaanbaatar | 1.48  (1.11-1.99) | 0.008 | 1.40  (1.07-1.82) | 0.014 | 1.39  (1.06-1.82) | 0.017 |
| **Education** | | | | | | |
| None | 1  (Reference) |  | - |  | - |  |
| Primary | 0.63  (0.39-1.03) | 0.065 | - |  | - |  |
| Secondary | 0.63  (0.42-0.96) | 0.031 | - |  | - |  |
| College ≤ | 0.69  (0.45-1.06) | 0.088 | - |  | - |  |
| **Monthly income (×1000 MNT)** | | | | | | |
| <100 | 1  (Reference) |  | - |  | 1  (Reference) |  |
| 100-<300 | 1.53  (1.15-2.04) | 0.004 | - |  | 1.52  (1.14-2.03) | 0.004 |
| 300-<500 | 1.41  (1.05-1.90) | 0.023 | - |  | 1.37  (1.02-1.84) | 0.037 |
| 500-<1000 | 1.29  (1.02-1.63) | 0.031 | - |  | 1.27  (1.01-1.59) | 0.040 |
| 1000 ≤ | 1.36  (1.06-1.76) | 0.016 | - |  | 1.34  (1.05-1.71) | 0.018 |
| **Currently drinking** | | | | | | |
| No | 1  (Reference) |  | 1  (Reference) |  | - |  |
| Yes | 1.13  (0.98-1.32) | 0.098 | 1.17  (1.02-1.35) | 0.028 | - |  |
| **Insufficient fruit and vegetable intake** | | | | | | |
| No | 1  (Reference) |  | 1  (Reference) |  | 1  (Reference) |  |
| Yes | 0.83  (0.71-0.96) | 0.016 | 0.83  (0.72-0.97) | 0.018 | 0.83  (0.71-0.96) | 0.014 |
| **Physical activity** | | | | | | |
| High | 1  (Reference) |  | 1  (Reference) |  | 1  (Reference) |  |
| Moderate | 1.38  (1.15-1.66) | <0.001 | 1.39  (1.16-1.65) | <0.001 | 1.39  (1.17-1.66) | <0.001 |
| Low | 1.77  (1.45-2.15) | <0.001 | 1.76  (1.45-2.12) | <0.001 | 1.78  (1.47-2.15) | <0.001 |
| **History of HT** | | | | | | |
| No | 1  (Reference) |  | 1  (Reference) |  | 1  (Reference) |  |
| Yes | 1.93  (1.66-2.24) | <0.001 | 1.98  (1.71-2.29) | <0.001 | 1.98  (1.71-2.29) | <0.001 |
| **History of DM** | | | | | | |
| No | 1  (Reference) |  | 1  (Reference) |  | 1  (Reference) |  |
| Yes | 2.22  (1.61-3.05) | <0.001 | 2.30  (1.68-3.15) | <0.001 | 2.29  (1.67-3.14) | <0.001 |
| **Body mass index** | | | | | | |
| Normal | 1  (Reference) |  | 1  (Reference) |  | 1  (Reference) |  |
| Underweight | 0.60  (0.29-1.27) | 0.180 | 0.61  (0.29-1.28) | 0.192 | 0.60  (0.29-1.26) | 0.178 |
| Overweight | 6.53  (5.46-7.80) | <0.001 | 6.47  (5.42-7.71) | <0.001 | 6.47  (5.42-7.73) | <0.001 |
| Obesity | 16.11  (13.19-19.67) | <0.001 | 15.78  (12.96-19.22) | <0.001 | 16.09  (13.19-19.62) | <0.001 |

MNT, Mongolian tugrik; HT, hypertension; DM, diabetes mellitus; AOR, adjusted odds ratio; CI, confidence interval.

Model 1: forced-entry, Model 2: forward-selection, Model 3: backward-selection.

Hosmer-Lemeshow test: P = 0.280 (Model 1), P = 0.567 (Model 2), and P = 0.389 (Model 3).

1 USD = 3,481.66 MNT on April 30, 2023.
