# Supplementary material for: Sex differences in the prevalence of metabolic syndrome and associated factors in the general population of Mongolia: A nationwide study
Source: PLoS One. 2024 Oct 23;19(10):e0311320. doi: 10.1371/journal.pone.0311320 (PMC11498733; doi:10.1371/journal.pone.0311320)
Supplement: S5 Table — (DOCX) [file pone.0311320.s005.docx]

**S5 Table. Factors associated with metabolic syndrome among men (N = 2,577).**

| **Variables** | **Model 1** | | **Model 2** | | **Model 3** | |
| --- | --- | --- | --- | --- | --- | --- |
|  | **AOR**  **(95% CI)** | **P-value** | **AOR**  **(95% CI)** | **P-value** | **AOR**  **(95% CI)** | **P-value** |
| **Age group (years)** | | | | | | |
| 18-29 | 1  (Reference) |  | 1  (Reference) |  | 1  (Reference) |  |
| 30-45 | 1.57  (1.06-2.33) | 0.025 | 1.68  (1.18-2.39) | 0.004 | 1.71  (1.20-2.44) | 0.003 |
| 45-69 | 1.96  (1.30-2.95) | 0.001 | 2.16  (1.51-3.08) | <0.001 | 2.17  (1.52-3.11) | <0.001 |
| **Ethnicity** | | | | | | |
| Khalkh | 1  (Reference) |  | - |  | 1  (Reference) |  |
| Kazak | 1.54  (0.75-3.17) | 0.237 | - |  | 1.48  (0.73-3.00) | 0.279 |
| Durvud | 0.86  (0.47-1.57) | 0.622 | - |  | 0.89  (0.49-1.61) | 0.701 |
| Buriad | 2.32  (1.13-4.80) | 0.023 | - |  | 2.30  (1.14-4.65) | 0.021 |
| Other | 1.40  (0.82-2.39) | 0.212 | - |  | 1.42  (0.84-2.40) | 0.194 |
| **Region** | | | | | | |
| Western | 1  (Reference) |  | - |  | 1  (Reference) |  |
| Eastern | 0.79  (0.45-1.39) | 0.418 | - |  | 0.86  (0.50-1.48) | 0.590 |
| Khangai | 0.77  (0.48-1.25) | 0.297 | - |  | 0.78  (0.49-1.26) | 0.311 |
| Central | 1.30  (0.79-2.15) | 0.299 | - |  | 1.28  (0.78-2.09) | 0.324 |
| Ulaanbaatar | 1.67  (1.02-2.71) | 0.040 | - |  | 1.60  (1.03-2.48) | 0.036 |
| **Education** | | | | | | |
| None | 1  (Reference) |  | - |  | - |  |
| Primary | 0.48  (0.23-1.00) | 0.049 | - |  | - |  |
| Secondary | 0.57  (0.31-1.08) | 0.085 | - |  | - |  |
| College ≤ | 0.53  (0.28-1.03) | 0.061 | - |  | - |  |
| **Monthly income (×1000 MNT)** | | | | | | |
| <100 | 1  (Reference) |  | - |  | - |  |
| 100-<300 | 1.65  (1.03-2.66) | 0.039 | - |  | - |  |
| 300-<500 | 1.52  (0.92-2.52) | 0.101 | - |  | - |  |
| 500-<1000 | 1.45  (1.00-2.10) | 0.052 | - |  | - |  |
| 1000 ≤ | 1.50  (1.00-2.24) | 0.048 | - |  | - |  |
| **Currently drinking** | | | | | | |
| No | 1  (Reference) |  | 1  (Reference) |  | 1  (Reference) |  |
| Yes | 1.30  (1.03-1.65) | 0.028 | 1.27  (1.01-1.59) | 0.037 | 1.28  (1.02-1.60) | 0.034 |
| **Physical activity** | | | | | | |
| High | 1  (Reference) |  | 1  (Reference) |  | 1  (Reference) |  |
| Moderate | 1.46  (1.10-1.95) | 0.010 | 1.41  (1.07-1.85) | 0.015 | 1.45  (1.10-1.92) | 0.009 |
| Low | 1.73  (1.26-2.36) | <0.001 | 1.69  (1.26-2.27) | <0.001 | 1.75  (1.30-2.35) | <0.001 |
| **History of HT** | | | | | | |
| No | 1  (Reference) |  | 1  (Reference) |  | 1  (Reference) |  |
| Yes | 1.85  (1.44-2.39) | <0.001 | 1.92  (1.50-2.46) | <0.001 | 1.92  (1.50-2.46) | <0.001 |
| **History of DM** | | | | | | |
| No | 1  (Reference) |  | 1  (Reference) |  | 1  (Reference) |  |
| Yes | 3.19  (1.79-5.66) | <0.001 | 3.44  (1.97-5.99) | <0.001 | 3.46  (1.98-6.06) | <0.001 |
| **Body mass index** | | | | | | |
| Normal | 1  (Reference) |  | 1  (Reference) |  | 1  (Reference) |  |
| Underweight | 0.92  (0.27-3.11) | 0.894 | 1.01  (0.30-3.39) | 0.991 | 0.94  (0.28-3.18) | 0.925 |
| Overweight | 16.98  (12.16-23.72) | <0.001 | 16.46  (11.86-22.83) | <0.001 | 16.51  (11.89-22.92) | <0.001 |
| Obesity | 56.10  (38.33-82.10) | <0.001 | 53.75  (37.06-77.95) | <0.001 | 54.07  (37.23-78.51) | <0.001 |

MNT, Mongolian tugrik; HT, hypertension; DM, diabetes mellitus; AOR, adjusted odds ratio; CI, confidence interval.

Model 1: forced-entry, Model 2: forward-selection, Model 3: backward-selection.

Hosmer-Lemeshow test: P = 0.607 (Model 1), P = 0.868 (Model 2), and P = 0.346 (Model 3).

1 USD = 3,481.66 MNT on April 30, 2023.
