# Supplementary material for: Sex differences in the prevalence of metabolic syndrome and associated factors in the general population of Mongolia: A nationwide study
Source: PLoS One. 2024 Oct 23;19(10):e0311320. doi: 10.1371/journal.pone.0311320 (PMC11498733; doi:10.1371/journal.pone.0311320)
Supplement: S6 Table — (DOCX) [file pone.0311320.s006.docx]

**S6 Table. Factors associated with metabolic syndrome among women (N = 3,188).**

| **Variables** | **Model 1** | | **Model 2** | | **Model 3** | |
| --- | --- | --- | --- | --- | --- | --- |
|  | **AOR**  **(95% CI)** | **P-value** | **AOR**  **(95% CI)** | **P-value** | **AOR**  **(95% CI)** | **P-value** |
| **Age group (years)** | | | | | | |
| 18-29 | 1  (Reference) |  | 1  (Reference) |  | 1  (Reference) |  |
| 30-45 | 1.48  (1.09-2.03) | 0.013 | 1.53  (1.15-2.03) | 0.004 | 1.53  (1.15-2.03) | 0.004 |
| 45-69 | 2.65  (1.94-3.63) | <0.001 | 2.73  (2.06-3.63) | <0.001 | 2.73  (2.06-3.63) | <0.001 |
| **Ethnicity** | | | | | | |
| Khalkh | 1  (Reference) |  | 1  (Reference) |  | 1  (Reference) |  |
| Kazak | 1.45  (0.77-2.74) | 0.254 | 1.35  (0.75-2.42) | 0.313 | 1.35  (0.75-2.42) | 0.313 |
| Durvud | 0.65  (0.40-1.06) | 0.084 | 0.57  (0.36-0.90) | 0.015 | 0.57  (0.36-0.90) | 0.015 |
| Buriad | 1.62  (0.89-2.93) | 0.111 | 1.51  (0.86-2.65) | 0.154 | 1.51  (0.86-2.65) | 0.154 |
| Other | 0.78  (0.48-1.25) | 0.295 | 0.66  (0.42-1.02) | 0.060 | 0.66  (0.42-1.02) | 0.060 |
| **Currently smoking** | | | | | | |
| No | 1  (Reference) |  | 1  (Reference) |  | 1  (Reference) |  |
| Yes | 1.55  (1.08-2.23) | 0.018 | 1.53  (1.07-2.18) | 0.019 | 1.53  (1.07-2.18) | 0.019 |
| **Insufficient fruit and vegetable intake** | | | | | | |
| No | 1  (Reference) |  | 1  (Reference) |  | 1  (Reference) |  |
| Yes | 0.82  (0.67-0.99) | 0.041 | 0.77  (0.64-0.93) | 0.006 | 0.77  (0.64-0.93) | 0.006 |
| **Physical activity** | | | | | | |
| High | 1  (Reference) |  | 1  (Reference) |  | 1  (Reference) |  |
| Moderate | 1.32  (1.04-1.68) | 0.025 | 1.36  (1.08-1.72) | 0.008 | 1.36  (1.08-1.72) | 0.008 |
| Low | 1.69  (1.30-2.20) | <0.001 | 1.75  (1.37-2.25) | <0.001 | 1.75  (1.37-2.25) | <0.001 |
| **History of HT** | | | | | | |
| No | 1  (Reference) |  | 1  (Reference) |  | 1  (Reference) |  |
| Yes | 1.99  (1.64-2.42) | <0.001 | 2.08  (1.73-2.51) | <0.001 | 2.08  (1.73-2.51) | <0.001 |
| **History of DM** | | | | | | |
| No | 1  (Reference) |  | 1  (Reference) |  | 1  (Reference) |  |
| Yes | 1.88  (1.27-2.80) | 0.002 | 1.89  (1.28-2.79) | 0.001 | 1.89  (1.28-2.79) | 0.001 |
| **Body mass index** | | | | | | |
| Normal | 1  (Reference) |  | 1  (Reference) |  | 1  (Reference) |  |
| Underweight | 0.50  (0.19-1.29) | 0.151 | 0.51  (0.20-1.31) | 0.161 | 0.51  (0.20-1.31) | 0.161 |
| Overweight | 3.63  (2.90-4.53) | <0.001 | 3.66  (2.94-4.56) | <0.001 | 3.66  (2.94-4.56) | <0.001 |
| Obesity | 7.92  (6.21-10.10) | <0.001 | 7.99  (6.28-10.16) | <0.001 | 7.99  (6.28-10.16) | <0.001 |

MNT, Mongolian tugrik; HT, hypertension; DM, diabetes mellitus; AOR, adjusted odds ratio; CI, confidence interval.

Model 1: forced-entry, Model 2: forward-selection, Model 3: backward-selection.

Hosmer-Lemeshow test: P = 0.430 (Model 1), P = 0.074 (Model 2), and P = 0.074 (Model 3).

1 USD = 3,481.66 MNT on April 30, 2023.
