# Supplementary material for: Sex differences in the prevalence of metabolic syndrome and associated factors in the general population of Mongolia: A nationwide study
Source: PLoS One. 2024 Oct 23;19(10):e0311320. doi: 10.1371/journal.pone.0311320 (PMC11498733; doi:10.1371/journal.pone.0311320)
Supplement: Attachment — Submitted filename: Response to reviewers.docx [file pone.0311320.s007.docx]

**Response to the editor’s and reviewers’ comments**

We would like to thank the editor and reviewers for reviewing our manuscript. We have revised the manuscript according to your comments, which were very helpful. The revised manuscript has been proofread by a native English speaker. The revisions have been completed and our responses are as follows.

**Journal Requirements:**

**1. Please ensure that your manuscript meets PLOS ONE's style requirements, including those for file naming. The PLOS ONE style templates can be found at**

[**https://journals.plos.org/plosone/s/file?id=wjVg/PLOSOne_formatting_sample_main_body.pdf**](https://journals.plos.org/plosone/s/file?id=wjVg/PLOSOne_formatting_sample_main_body.pdf) **and**

[**https://journals.plos.org/plosone/s/file?id=ba62/PLOSOne_formatting_sample_title_authors_affiliations.pdf**](https://journals.plos.org/plosone/s/file?id=ba62/PLOSOne_formatting_sample_title_authors_affiliations.pdf)

We have carefully revised our manuscript to meet PLOS ONE’s style requirements including the file names.

**2.** **Note from Emily Chenette, Editor in Chief of PLOS ONE, and Iain Hrynaszkiewicz, Director of Open Research Solutions at PLOS: Did you know that depositing data in a repository is associated with up to a 25% citation advantage (**[**https://doi.org/10.1371/journal.pone.0230416**](https://doi.org/10.1371/journal.pone.0230416)**)? If you’ve not already done so, consider depositing your raw data in a repository to ensure your work is read, appreciated and cited by the largest possible audience. You’ll also earn an Accessible Data icon on your published paper if you deposit your data in any participating repository (**[**https://plos.org/open-science/open-data/#accessible-data**](https://plos.org/open-science/open-data/#accessible-data)**).**

Thank you for your suggestion. The data that support the findings of this study are available at the WHO NCD Microdata Repository (<https://extranet.who.int/ncdsmicrodata/index.php/catalog>).

**3. Please review your reference list to ensure that it is complete and correct. If you have cited papers that have been retracted, please include the rationale for doing so in the manuscript text, or remove these references and replace them with relevant current references. Any changes to the reference list should be mentioned in the rebuttal letter that accompanies your revised manuscript. If you need to cite a retracted article, indicate the article’s retracted status in the References list and also include a citation and full reference for the retraction notice.**

We have revised the references according to the comment No. 12 by Reviewer 1. The below table shows how we have revised references and reference numbers. We used EndNote (a software for reference manager) and revision of references and reference numbers cannot be retracted using the function of Track Change. Therefore, we have used red color for revised reference number and revised references in the reference section.

We are very sorry that the reference style did not follow the journal style. We have checked the reference style on the journal site and revised the reference style in the manuscript.

| Ref. | Original | Revision |
| --- | --- | --- |
| 1 | Deepa M, 2007 | The IDF consensus worldwide definition, 2006 |
| 5 | Ford ES, 2005 | Yakoob MY, 2016 |
| 7 | The IDF consensus worldwide definition, 2006 | Ref. number changed to 1. |
| Ref. 8-21 | | Ref. 7-20 |
| 22 | NCEP expert panel on detection, evaluation,… , 2002 | Ref. number changed to 26 |
| 23 | National Center for Public Health, 2020 | Ref. number changed to 21 |
| 24 | Schneider JT, 2006 | (Ref. 22) Iwasa T, 2023. |
| 25 | Regitz-Zagrosek C, 2007 | (Ref. 23) Meloni A, 2023 |
| 26 | Cornier MA, 2008 | (Ref. 24) Kautzky-Willer A, 2023 |
| 27 | Kuk JL, 2010 | (Ref. 25) Tramunt B, 2020 |
| 28 | Morales DD, 2008 | Changed to appropriate reference (Ref. 18) |
| 29 | Zago Z, 2004 | (Ref. 27) Li H, 2021 |
| Ref. 30-36 | | Ref. 28-34 |
| 37 | Fujita N, 2011 | (Ref. 35) Hirakawa M, 2015 |
| 38 | Alkerwi A, 2009 | Erased due to Ref. 34 and Ref. 35 are enough for the sentence. |
| 39 | Yoon YS, 2004 |  |
| Ref. 40-43 | | Ref. 36-39 |
| 44 | Halton TL, 2006 | (Ref. 40) Muraki I, 2016 |
| 45 | Mozaffarian D, 2011 | (Ref. 41) Hojati A, 2024 |
| 46 | Beulens JW, 2007 | (Ref. 42) Fung TT, 2016 |

**Reviewer #1:**

1. **Title; It's better to mention STEPS or "a nation-wide study" instead of "a cross-sectional study."**

According to the reviewer’s comment and advice of the English proofreader, we have revised the title from “Sex differences in the prevalence and factors associated with metabolic syndrome in the general population of Mongolia: A cross-sectional study” to “Sex differences in the prevalence of metabolic syndrome and associated factors in the general population of Mongolia: A nationwide study.”

2. **Abstract, and methods of main manuscript: I suggest exclude data of individuals <18 years old. They categorized as adolescents. So, interpretation of the findings in them can be different from other age groups. If you prefer to remain their data, I think it's better to analysis their data separately from other age-groups.**

We have excluded participants who were younger than 18 years old and the revised manuscript includes 5,695 participants who were 18 years old or older.

3. **Introduction; please update Ref.7.**

Line 50: The reviewer suggested to update the reference of the definition and criteria of metabolic syndrome, but we think that the reference in our manuscript is the latest version (International Diabetes Federation. The IDF consensus worldwide definition of the metabolic syndrome. Brussels, Belgium: International Diabetes Federation; 2006).

The reference number in Line 50 has been changed from 7 to 1, because we have changed the reference for the first sentence of the introduction section from “Deepa et al, Diabetes Metab Res Rev. 2007” to “The IDF consensus worldwide definition of the metabolic syndrome, 2006.” The first sentence explains about component of metabolic syndrome; therefore, “The IDF consensus worldwide definition of the metabolic syndrome, 2006” is more appropriate than the previous Ref. 1 (Deepa et al, Diabetes Metab Res Rev. 2007).

4. **Insert date of the study in line 69**

Line 69: We have added the year (2019) of the study as follows, “58.0% in adults ≥20 years old in a rural area in 2019.”

5. **Method; please insert Ethical Committee approval code.**

Line 148: We have added the ethical approval number #53 in the sentence.

6. **Results; It is missed expansion of some abbreviated words in the first use, such as HEC.**

Line 175: HEC was our mistake and it has been revised to “HCE. We have checked all abbreviations and added the full spelling for an abbreviation when it is used for the first time.

7. **Please insert data of "central obesity" in all Tables.**

We have added “abdominal obesity (=central obesity)” in Tables 2 and 3.

8. **For all tables, insert Statistical analysis test in the legend.**

We have added statistical analysis test in the footnote of Tables 2 and 3.

9. **Please merge tables 3, and 4.**

We have merged Tables 3 and 4 into one table (Table 3).

10. **Insert additional Tables, based on "urban/rural", "BMI", and "central obesity", separately.**

We have made three tables based on residence (rural and urban), BMI (normal, underweight, overweight, and obesity), and abdominal obesity (yes and no) and put them as S1-3 Tables in supporting information file. Supporting information captions has been added at the end of the manuscript file.

11. **Discussion; The first paragraph should address the main findings. So, description of results and comparison with other studies should start from the second paragraph.**

Lines 270-277: We have revised the first paragraph of the discussion section and addressed the main findings as follows, “In this study, the prevalence of MetS was 37.4% in the general adult population of Mongolia. The MetS prevalence was significantly higher in women than men. The most prevalent MetS component was abdominal obesity in women but elevated FBG in men. Factors associated with MetS among both men and women were older age, low and moderate PA level, history of HT and DM, high BMI (overweight and obesity), and abdominal obesity. Ethnicity, education, income, current alcohol consumption were the associated factors only among men and current smoking and sufficient fruit and vegetable intake were only among women. These results showed that there were differences in the MetS prevalence, MetS component, and the associated factors among sexes.”

12. **Ref.; nearly half of the Ref. are published on more than 10 years ago. Please update.**

We have revised 10 references that were published more than 10 years ago to more recent papers (published in 2015 or after). According to revision of the manuscript, reference numbers have been revised. The below table shows how we have revised references. We used EndNote (a software for reference manager) and revision of references and reference numbers cannot be retracted using the function of Track Change. Therefore, we have used red color for revised reference number and revised references in the reference section.

| Ref. | Original | Revision |
| --- | --- | --- |
| 1 | Deepa M, 2007 | The IDF consensus worldwide definition, 2006 |
| 5 | Ford ES, 2005 | Yakoob MY, 2016 |
| 7 | The IDF consensus worldwide definition, 2006 | Ref. number changed to 1. |
| Ref. 8-21 | | Ref. 7-20 |
| 22 | NCEP expert panel on detection, evaluation,… , 2002 | Ref. number changed to 26 |
| 23 | National Center for Public Health, 2020 | Ref. number changed to 21 |
| 24 | Schneider JT, 2006 | (Ref. 22) Iwasa T, 2023. |
| 25 | Regitz-Zagrosek C, 2007 | (Ref. 23) Meloni A, 2023 |
| 26 | Cornier MA, 2008 | (Ref. 24) Kautzky-Willer A, 2023 |
| 27 | Kuk JL, 2010 | (Ref. 25) Tramunt B, 2020 |
| 28 | Morales DD, 2008 | Changed to appropriate reference (Ref. 18) |
| 29 | Zago Z, 2004 | (Ref. 27) Li H, 2021 |
| Ref. 30-36 | | Ref. 28-34 |
| 37 | Fujita N, 2011 | (Ref. 35) Hirakawa M, 2015 |
| 38 | Alkerwi A, 2009 | Erased due to Ref. 34 and Ref. 35 are enough for the sentence. |
| 39 | Yoon YS, 2004 |  |
| Ref. 40-43 | | Ref. 36-39 |
| 44 | Halton TL, 2006 | (Ref. 40) Muraki I, 2016 |
| 45 | Mozaffarian D, 2011 | (Ref. 41) Hojati A, 2024 |
| 46 | Beulens JW, 2007 | (Ref. 42) Fung TT, 2016 |

13. **Consider language editing for whole manuscript. In some sentences is observed numerous grammatical errors.**

We have asked a native English speaker who has a PhD to proofread the manuscript and we have revised our manuscript accordingly.

**Reviewer #2:**

14. **Criteria used for MetS are described based on (IDF) and Adult Treatment Panel (ATP) for estimating the prevalence of MetS, but in other parts only IDF criteria are used. I think ATP criteria may not be necessary. The paper just can only use IDF critera.**

We have erased the explanation about the criteria of ATP III and results based on the ATP III criteria in our manuscript and figures.

15. **Table 2 provides no more information compared to Table 3. It could be potentially removed.**

This study focuses on differences between men and women in terms of metabolic syndrome and Table 3 shows the differences in factors associated with metabolic syndrome between the sexes. Table 2 shows the differences in the characteristics between male and female participants, and the characteristics differences were used to explain why differences were found in the prevalence of metabolic syndrome and associated factors between the sexes in the discussion section. Therefore, we would like to keep Table 2 in our manuscript.

16. **Table 3, is the adjusted p-value calculated after adjustment with all variables? (more than 15 variables are introduced)**

Lines 139-142: We included all variables in Table 3 in multivariable logistic regression analyses. Before performing multivariable analysis, we confirmed there was no absence of a strong correlation among the independent variables. We also confirmed that the result of Hosmer-Lemeshow test was P > 0.05 in each model. We have added the following sentences in the method section, “Multivariable logistic regression analysis was performed on MetS using all the variables other than abdominal obesity. A correlation matrix was created to confirm the absence of strong correlation (γ > 0.80) among the independent variables. The result of the Hosmer-Lemeshow test of each model was P > 0.05. “

Lines 239-243: We have also added the result of Hosmer-Lemeshow test in the footnote of Table 3 as follows, “^d^Multivariable logistic regression analysis was performed including all variables in the table other than abdominal obesity (Hosmer-Lemeshow test: P = 0.280).

^e^Multivariable logistic regression analysis was performed including all variables in the table other than sex and abdominal obesity (Hosmer-Lemeshow test: P = 0.607 [men] and P = 0.430 [women]).”
