# Supplementary material for: Sex differences in the prevalence of metabolic syndrome and associated factors in the general population of Mongolia: A nationwide study
Source: PLoS One. 2024 Oct 23;19(10):e0311320. doi: 10.1371/journal.pone.0311320 (PMC11498733; doi:10.1371/journal.pone.0311320)
Supplement: Attachment — Submitted filename: Response to Reviewers.docx [file pone.0311320.s008.docx]

**Response to the editor’s and reviewers’ comments**

We would like to thank the editor and reviewers for reviewing our manuscript. We have revised the manuscript according to your comments, which were very helpful. The revised manuscript has been proofread by a native English speaker. The revisions have been completed and our responses are as follows.

**Journal Requirements:**

**1. Please review your reference list to ensure that it is complete and correct. If you have cited papers that have been retracted, please include the rationale for doing so in the manuscript text, or remove these references and replace them with relevant current references. Any changes to the reference list should be mentioned in the rebuttal letter that accompanies your revised manuscript. If you need to cite a retracted article, indicate the article’s retracted status in the References list and also include a citation and full reference for the retraction notice.**

We have added a new reference (Ref 21) according to the comment by Reviewer 1. In the manuscript, reference numbers of 21-42 have been revised to 22-43. We used EndNote (a software for reference manager) and revision of references and reference numbers cannot be retracted using the function of Track Change. Therefore, we have used red color for revised reference numbers and revised references in the reference section.

**Additional Editor Comments:**

**2. I am ready to accept the paper, provided it is revised with English Editing.**
The revised manuscript has been English proofread by a native English speaker. Therefore, there are many parts that have been revised according to the advice of the English proofreader.

**Reviewer #1:**

**3. All comments are adequately addressed, just please re-check sum of new estimations for each column. For example, sum of HTN data is incorrect.**

Lines 125-130, Table 2, and S1-3 Tables: Our study used the data from 5,695 participants who had no missing values for variables related to the MetS criteria, but some missing values for other variables. We have added the explanation about the missing values in the methods section as follows, “The total number of STEPS participants was 6,654 but 959 participants were excluded due to missing values on MetS measurement data. Finally, the data of 5,695 participants were analyzed in this study. There were missing values about ethnicity (n = 26), education (n = 1), marital status (n = 7), employment (n = 120), fruit and vegetable intake (n = 240), physical activity (n = 88), and body mass index (n = 1). The missing values were treated by multiple imputation method [21].” We have also added the number of participants for variables that had missing data in Table 2 and S1-3 Tables.

Line 112: We have revised the sentence as follow, “….5,695 adult participants of the STEPS who were 18‒69 years old and whose data had no missing values for variables related to the MetS criteria.”

We are very sorry that there were mistakes of numbers and percentages in tables. We have carefully checked all numbers and percentages in all tables and revised them correctly.

**Reviewer #3:**

**4. Dietary assessment was perhaps the only limitation from this study where diet could have also played a vital role in the analysis of the reported study.**

Lines 361-362: We have added a sentence in the paragraph of limitations as follow, “Dietary assessment was limited in this study, although diet may also play an important role in the development of MetS.”

**5. Correlation tests and Regression models could have been tried and tested for a more analytical statistical interpretation.**

S4-6 Tables: According to the comment, we have performed two additional models of multivariate logistic regression, namely stepwise forward-selection and backward-selection. We have added three tables to show factors associated with MetS among all participants (S4 Table), men (S5 Table), and women (S6 Table) using the three models.

Lines 143-147: In the methods section, we have added as follows, “In multivariable logistic regression analysis on MetS, three models were performed. In Model 1 (the forced-entry method), all the variables other than abdominal obesity were included. In Models 2 and 3 (the forward and backward stepwise selection method), variables were added when the P-value was less than 0.05 and removed when the P-value was over 0.1.“

Lines 272-279: In the results section, we have added as follows, “The results of multivariate logistic regression analyses for MetS among all participants, men, and women using three models are shown in S4-6 Tables. Older age (≥ 30 years old), low and moderate PA level, history of HT and DM, high BMI (overweight and obesity) were associated with MetS among all participants, men, and women in all three models. In addition, factors associated with MetS in the three models were Buriad ethnicity, residence in the central region and Ulaanbaatar, sufficient fruit and vegetable intake among all participants, current alcohol drinkers among men, and current smokers and sufficient fruit and vegetable intake among women.”
